# Neural correlates of successful emotion recognition in healthy elderly: a multimodal imaging study

**DOI:** 10.1093/scan/nsad058

**Published:** 2023-10-26

**Authors:** Isabella Orlando, Carlo Ricci, Ludovica Griffanti, Nicola Filippini

**Affiliations:** Department of Psychology, Salesian Pontifical University of Rome, Rome 00139, Italy; Department of Psychology, Salesian Pontifical University of Rome, Rome 00139, Italy; Department of Psychology, Walden Institute of Rome, Rome 00186, Italy; Wellcome Centre for Integrative Neuroimaging, Oxford Centre for Human Brain Activity, Department of Psychiatry, University of Oxford, Oxford OX3 7JX, UK; Wellcome Centre for Integrative Neuroimaging, Oxford Centre for Functional MRI of the Brain, Nuffield Department of Clinical Neurosciences, University of Oxford, Oxford OX3 9DU, UK; IRCCS San Camillo Hospital, Venice 30126, Italy

**Keywords:** emotion recognition, ageing, functional connectivity, structural MRI, diffusion MRI

## Abstract

The ageing process is associated with reduced emotional recognition (ER) performance. The ER ability is an essential part of non-verbal communication, and its role is crucial for proper social functioning. Here, using the ‘Cambridge Centre for Ageing and Neuroscience cohort sample’, we investigated when ER, measured using a facial emotion recognition test, begins to consistently decrease along the lifespan. Moreover, using structural and functional MRI data, we identified the neural correlates associated with ER maintenance in the age groups showing early signs of ER decline (*N* = 283; age range: 58–89 years). The ER performance was positively correlated with greater volume in the superior parietal lobule, higher white matter integrity in the corpus callosum and greater functional connectivity in the mid-cingulate area. Our results suggest that higher ER accuracy in older people is associated with preserved gray and white matter volumes in cognitive or interconnecting areas, subserving brain regions directly involved in emotional processing.

## Introduction

Studies aimed at investigating changes associated with the ageing process have arisen over the past years, allowing a more thorough understanding of modifications occurring when we age. Up to date, research has largely focused on investigating age-related cognitive changes. Several studies have shown that impairments in cognitive skills may affect the quality-of-life (Harada *et al.*, 2013) and predict conversion to neurodegenerative disorders (Masur *et al.*, 1994; Wearn *et al.*, 2020). Advanced imaging techniques have been effective in identifying structural and functional early markers reflecting resilience against and vulnerability to the development of late-life pathologies, and/or compensatory mechanisms to counteract cognitive decline (Anaturk *et al.*, 2021). However, some other aspects, such as emotional functioning, have not yet been so deeply investigated, despite their importance in everyday life.

Investigation on emotion is a complex and multifaceted field. It encompasses aspects such as emotional experience/expression, emotion regulation/control, emotion expression/recognition and emotional memory/attention (Kremer and den Uijl, 2016). In this study, we focused on emotion recognition (ER), in particular on the age-related changes associated with recognizing others’ emotions. This is an essential part of non-verbal communication (Kuenecke *et al.*, 2017), and it is important not only to ensure proper emotional functioning, but also for its crucial role in social functioning (Sze *et al.*, 2012). Poor ER has been associated with reduced interpersonal well-being and social interest (Carton *et al.*, 1999), potentially leading to social isolation (Strijkert *et al.*, 2022). Moreover, reduced ER accuracy in older adults may reflect impending depressive- and anxiety-related symptoms (Orgeta, 2011), which are increasingly common in an older population (Cheng *et al.*, 2021), and in certain cases they may even precede the onset of neurodegenerative disorders (Ochi and Midorikawa, 2021).

Behavioral studies have shown that ER is prone to age-related changes (Sullivan and Ruffman, 2004; Ruffman *et al.*, 2008). A meta-analysis, including studies using a research design based on ‘younger vs older’ healthy adults’ comparison, has shown a consistent reduction in ER performance associated with age for a variety of emotions (anger, fear, disgust, happiness, sadness, surprise) (Ruffman *et al.*, 2008). These results were confirmed by a more recent and independent meta-analysis (Gonçalves *et al.*, 2018). Three studies have evaluated age-related changes across the entire lifespan and all of them have reported significant age-related-reduced performance. Calder et al., using a facial recognition task, reported a negative effect of age on fear and anger stimuli (Calder *et al.*, 2003). Isaacowitz et al. found a consistent age effect, with some variation on the type of emotion affected, based on the stimulus presentation modality used. Lexical stimuli were associated with age groups differences in accuracy for all investigated emotions except fear, whereas facial stimuli were related to age groups differences in accuracy for anger, disgust, fear, and happiness (Isaacowitz *et al.*, 2007). Most recently, Malykhin et al. found significant age-related reduction in accuracy for happiness (Malykhin *et al.*, 2023).

An important limitation in the field is that the neural correlates associated with successful/unsuccessful ER with ageing have been scarcely investigated. This is relevant because, although behavioral measures are good indicators of changes in emotional functioning, neuroimaging techniques may be more sensitive and informative at identifying those specific brain regions associated with maintenance/decline in ER ability, unravelling the specific pathways influenced by the effect of age. Up to date, imaging studies performed to identify associations between neural changes and ER performance in older adults have largely focused on pathology (Baggio *et al.*, 2012; Park *et al.*, 2017; Rigon *et al.*, 2017). Very few studies have investigated age-related ER changes and neurophysiological measures in healthy adults. Wada et al. compared small groups of younger and older subjects and, using a region-of-interest (ROI) approach, they showed that the right supramarginal gyrus volume was associated with ER maintenance (Wada *et al.*, 2021). Thus far, to the best of our knowledge, only Malykhin et al. tested for an association between age-related reductions in ER and neurophysiological correlates (amygdala volume) on a large healthy cohort across the lifespan, but found no significant effect (Malykhin *et al.*, 2023).

In this study, we investigated age-related changes in ER accuracy and its association with magnetic resonance imaging (MRI) measures (both structural and functional), on data collected for the ‘The Cambridge Centre for Ageing and Neuroscience (Cam-CAN)’ project (Shafto *et al.*, 2014; Taylor *et al.*, 2017), with two specific aims. First, to identify when differences in ER performance start to consistently appear along the lifespan. Secondly, across the age groups showing the first signs of ER decline, to pinpoint those brain regions associated with successful ER processing. Understanding the neural correlates associated with age-related changes in ER, may reveal useful markers to detect the brain features and mechanisms associated with ER maintenance. Moreover, it may provide essential insights into the biological underpinnings that facilitate socially appropriate behavior and interpersonal well-being in older people. Ultimately, this can be potentially useful to identify those subjects at increased risk of developing future neurological and mental health conditions (Cotter *et al.*, 2018; Gonzalez-Alcaide *et al.*, 2021).

## Materials and methods

### Study sample

All participants included in this study were taken from the publicly available database Cam-CAN (available at http://www.mrc-cbu.cam.ac.uk/datasets/camcan/) (Shafto *et al.*, 2014; Taylor *et al.*, 2017). Data collection was performed in accordance with the Declaration of Helsinki, approved by the local ethics committee and all participants provided written informed consent prior to data acquisition for the study.

For this study, we selected subjects included in Stage II (‘CC700’ phase) of the Cam-CAN dataset. For the first research question, all participants with recorded information for age, sex, performance at the ER test and anatomical MRI, to exclude gross abnormalities in grey matter (GM) structures, were included in the behavioural analysis. The ER performance was defined as the sum of the scores across all the emotions employed in the facial ER task and was used as a global measure. For a full description of the task, see Section ‘Study sample’.

The characteristics of the study sample are summarized in Table 1.

**Table 1.** Overview of the sample (*N* = 637) characteristics and behavioral measures. Values denote mean (±SD), N reflects the number of participants and % denotes the percentage of participants. R, AD, L refers to right-handed, ambidextrous and left-handed participants, respectively

Sample characteristics	Group 1 [18–27 years]	Group 2 [28–37 years]	Group 3 [38–47 years]	Group 4 [48–57 years]	Group 5 [58–67 years]	Group 6 [68–77 years]	Group 7 [78+ years]
Socio-demographics							
Number of participants	50	101	101	96	99	99	91
Age	23.82(±2.96)	33.20(±3.00)	43.26(±2.92)	52.91(±3.01)	62.98(±2.86)	72.52(±2.93)	81.53(±2.68)
Sex (*N*, % female)	27 (54)	52 (51.5)	51 (50.5)	50 (52)	50 (52)	51 (51.5)	43 (47.3)
Handedness	74.12(±48.64)	81.23(±44.95)	74.63(±54.16)	78.06(±51.81)	74.88(±55.75)	81.55(±47.81)	87.45(±34.46)
R/AD/L (N)	46/0/4	93/1/7	90/1/10	86/2/8	86/4/9	91/2/6	85/3/3
ER test							
Total score	109.26(±8.18)	109.28(±9.07)	108.73±(8.91)	107.98±(7.78)	104.55±(11.49)	97.30±(15.16)	93.21±(15.11)
Reaction time (in ms)	2356(±516)	2293(±524)	2308(±567)	2240(±416)	2406(±503)	2646(±603)	2904(±609)
Cognitive scores							
MMSE score	29.18(±1.32)	29.51(±0.92)	29.09(±1.14)	29.20(±1.09)	29.01(±1.11)	28.59(±1.27)	28.95(±1.26)

For the second research question, only participants within the age groups showing decline in ER were selected for the subsequent analysis of MRI correlates of successful ER.

### ER test

The facial ER evaluation was based on morphed (blended) images adapted from the Ekman and Friesen original work (Ekman and Friesen, 1978). Facial emotion included in the test represented the following emotions: happy, sadness, anger, fear, disgust and surprise. Six expression pairs, happiness–surprise, surprise–fear, fear–sadness, sadness–disgust, disgust–anger, anger–happiness were presented as stimuli. Each pair contained the following percentages of the two emotional expressions, 90–10%, 70–30%, 50–50%, 30–70% and 10–90%. Performance was based only on trials where the morphed image was biased 70% or 90% towards one expression, giving a total accuracy maxima of 20 for each of the six expressions and 120 for the entire dataset. Participants were asked to select the right response among a list reporting, as potential choices, the six basic emotions. A brief practice test preceded the real experiment, which took ∼20 min. Both performance and reaction times were recorded. See Calder *et al.* (1996) and Shafto *et al.* (2014) for a more detailed description of the test. Here, to evaluate age-related ER variations, we have used the sum of the performance in all the studied emotions. The rationale for this choice was because previous studies had reported inconsistent results on which specific emotion was mostly affected by age (Calder *et al.*, 2003; Isaacowitz *et al.*, 2007; Malykhin *et al.*, 2023). Moreover, meta-analyses had consistently shown an overall reduction in ER scores in older subjects relative to younger participants (Ruffman *et al.*, 2008; Gonçalves *et al.*, 2018), and therefore we did not want to be biased towards a specific emotion, but our aim was to provide a comprehensive evaluation of the basic emotional spectrum. The ER test was performed outside the scanner.

### MRI data acquisition

MRI scans were performed at the Medical Research Council-Cognition and Brain Sciences Unit (MRC-CBSU) on a Siemens 3 Tesla (3T) TIM trio System (Siemens Healthcare GmbH, Erlangen, Germany) equipped with a 32-channel receiver head coil. Briefly, the data used in this study included both structural (anatomical and diffusion MRI) and resting state functional MRI (rs-fMRI) scans.

The anatomical scans were acquired using a 3D T1-weighted (T1w) magnetization prepared rapid gradient echo (MPRAGE) sequence with the following parameters: Repetition Time (TR) = 2250 ms; Echo Time (TE) = 2.99 ms; Inversion Time (TI) = 900 ms; flip angle = 9°; field of view (FOV) = 256 mm × 240 mm × 192 mm; voxel size = 1 mm isotropic; GeneRalized Autocalibrating Partial Parallel Acquisition (GRAPPA) acceleration factor = 2; acquisition time of 4 min and 32 s.

The diffusion-weighted images (DWIs) were acquired with a twice-refocused spin-echo sequence, with 30 diffusion gradient directions for each of two *b*-values: 1000 and 2000 s/mm^2^ and three images acquired with a *b*-value of 0. Other MRI parameters: TR = 9100 ms, TE = 104 ms, voxel size = 2 mm isotropic, FOV =192 mm × 192 mm, 66 axial slices, number of averages = 1; acquisition time of 10 min and 2 s.

The rs-fMRI images were acquired using a Gradient-Echo Echo-Planar Imaging (EPI) sequence. MRI parameters: TR = 1970 ms; TE =30 ms; flip angle =78°; FOV =192 mm × 192 mm; voxel-size =3 mm × 3 mm × 4.44 mm. Acquisition time of 8 min and 40 s, for a total number of 261 volumes acquired. During the rs-fMRI run, participants were instructed to rest with their eyes shut.

Further details about the MRI protocol can be found in Shafto *et al.* (2014) and Taylor *et al.* (2017).

### Behavioural analysis (*‘Changes in the emotion recognition performance across the lifespan’)*

ER change associated with the ageing process was first investigated across the entire lifespan (18- to 89-years old) in order to identify the first age-group in our sample group showing decline in the ER assessment. Participants were organized in decades as previously shown in other studies (Calder *et al.*, 2003; Isaacowitz *et al.*, 2007; Malykhin *et al.*, 2023). Univariate analysis of variance (ANOVA) test was used with the global ER score as dependent variable and all participants divided in decades, resulting in a total number of seven study groups, as group factor (submitted to polynomial contrast). Statistical analysis was carried out using SPSS software (SPSS, Inc., Version 28) and Games–Howell correction was applied for post-hoc comparisons.

### MRI analysis (*‘Neural correlates of successful emotion recognition in older participants’)*

#### MRI data processing

Data analysis was carried out using FSL tools (Smith *et al.*, 2004) and Mrtrix3 software (Tournier *et al.*, 2019) for part of DWI image pre-processing.

*Anatomical scans:* Pre-processing for structural images included the following steps: (i) re-orienting images to the standard (MNI) template, (ii) bias field correction, (iii) brain extraction and (iv) brain tissues segmentation using FMRIB’s Automated Segmentation Tool (FAST) that allows extracting global measures of total GM, white matter (WM) and cerebrospinal fluid (CSF). Whole-brain analysis was carried out using a voxel-based morphometry-style analysis (FSL-VBM) (Douaud *et al.*, 2007). Brain extraction and tissue-type segmentation were performed and resulting GM partial volume images were aligned to standard space using first linear (FLIRT) and then non-linear (FNIRT) registration tools. A study-specific template was created. Images were averaged, modulated and smoothed with an isotropic Gaussian kernel of 5 mm Full-Width at Half Max (FWHM) and the GM images were re-registered to the study-specific template, including modulation by the warp field Jacobian. Finally, voxel-wise general linear model (GLM, please see Section ‘MRI Statistical analysis - GLM model (variable of interest and nuisance covariates)’ for a full description of the model) was applied using permutation-based non-parametric testing (5000 permutations) (Nichols and Holmes, 2002) and Threshold-Free Cluster Enhancement (TFCE) (Smith and Nichols, 2009) to assess voxel-wise associations with the variable of interest. Family Wise Error (FWE)-corrected cluster significance threshold of *P* < 0.05 was applied to the suprathreshold clusters.

*DWI scans:* pre-processing steps for each subject data consisted of noise-level estimation and denoising (*dwidenoise*), Gibbs ringing artifacts removal (*mrdegibbs*), and motion and eddy current-induced distortion correction (*dwifslpreproc*). Fractional anisotropy (FA), mean diffusivity (MD), axial diffusivity (AD) and radial diffusivity (RD) maps were generated using DTIFit, part of FMRIB’s Diffusion Toolbox, that fits a diffusion tensor model at each voxel (Basser *et al.*, 1994). The FA output images were used as input for Tract-Based Spatial Statistics, a voxel-wise approach for analysis of FA data (Smith *et al.*, 2006). All subjects’ FA data were aligned into a common space using FMRIB’s Non-linear Image Registration Tool (FNIRT). The mean FA image was generated and thinned to create a mean FA skeleton, which represents the centres of all tracts common to the group. Each subject’s aligned FA data were then projected onto this skeleton and the resulting data fed into voxel-wise GLM cross-subject statistics. Voxel-wise GLM was applied using permutation-based non-parametric testing (5000 permutations) (Nichols and Holmes, 2002) and TFCE (Smith and Nichols, 2009) to assess voxel-wise associations with the variable of interest. FWE-corrected cluster significance threshold of *P* < 0.05 was applied to the suprathreshold clusters.

*Resting fMRI (rs-fMRI) scans:* data pre-processing consisted of motion correction, brain extraction, Gaussian kernel smoothing of FWHM of 5 mm, high-pass temporal filtering with a cut-off of 100 s (0.01 Hz), and it was carried out using first-level fMRI Expert Analysis Tool (FEAT) v. 6.00 (Woolrich *et al.*, 2001). FMRI volumes were registered to the individual’s structural scan and standard space images using both FLIRT and FNIRT registration tools, then optimized using boundary-based-registration approach (Greve and Fischl, 2009). In order to denoise functional images from the spurious signal and increase the possibility of identifying markers of effective connectivity, FMRIB’s ICA-based X-noiseifier (FIX) was applied (Griffanti *et al.*, 2014) and a training dataset specifically developed on our data. A seed-based connectivity analysis approach was used to investigate functional connectivity (FC) association with ER performance across the study participants. The peak of brain regions showing significant association between GM and ER performance was chosen as region of interest (ROI), whereas masks drawn within WM and CSF areas were used as nuisance regressors, to further remove spurious signal. For the first-level analysis (individual level), the mean time series of the ROIs were extracted for each participant, and the FC maps were obtained by calculating the temporal correlation coefficients between the mean time series of the seed regions and the time series of each voxel within the whole brain. The higher level (group level) analysis was carried out using FMRIB’s local analysis of mixed effects (FLAME) (Woolrich *et al.*, 2004) using a GLM including the variable of interest and the nuisance variables, in the same fashion as performed for the anatomical and DWI data analyses. The derived *Z*-statistic images were thresholded using clusters determined by *Z* > 3.1, and a FWE cluster significance threshold of *P* < 0.05 applied to the suprathreshold clusters.

#### MRI Statistical analysis—GLM model (variable of interest and nuisance covariates)

The global score at the ER evaluation was included as covariate of interest for the GLM analysis using MRI data. Other variables were included in the model as covariates of no interest (nuisance variables) in order to account for potential confounding effects influencing our results. Nuisance variables included: age, sex, handedness (assessed by the Edinburgh Handedness Inventory, with a score ranging from −100 to 100) (Oldfield, 1971), Mini Mental State Examination (MMSE) score (Folstein *et al.*, 1975), anxiety and depression scores (assessed by the Hospital Anxiety and Depression Scale—HADS) (Zigmond and Snaith, 1983), reaction time at the ER test, education level (expressed as a categorical variable based on qualification level, 0 = no degree, 1 = O/GCSE levels or equivalent, 2 = A levels or equivalent, 3 = NVQ, HND, HNC or other professional qualification, 4 = CSE or university and entered in the model using dummy coding) and type of gradient coil used during MRI acquisition, as the coil failed just before the first 100 scans. For the Edinburgh Handedness Inventory, a cut-off score ≥ 50 indicated righthandedness; < 50 to > − 50 indicated ambidextrous handedness; ≤ − 50 indicated left-handedness (Dragovic, 2004). Anxiety and depression scores were added as nuisance variables, as previous studies showed that they were associated with changes in brain imaging measures (Oathes *et al.*, 2015).

Given the heterogeneity of the measures, variable of interest and nuisances data were normalized before being included in the model.

## Results

### Changes in the emotion recognition performance across the lifespan

**Fig. 1.**
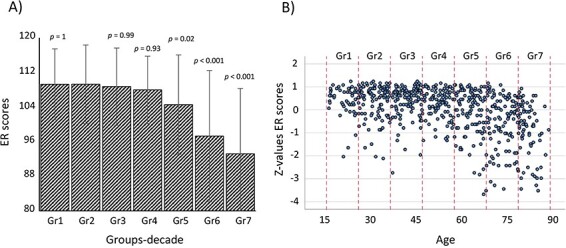
Emotion recognition (ER) score changes across the lifespan for the 637 subjects. (A) Bar charts reporting mean (±SD) on ER scores, and *P*-values (Games–Howell corrected) for the seven study groups across the lifespan (18- to 89-years old). (B) Scatter plot of individual ER scores (converted in *Z*-values) against age. Dotted lines are used to sub-divide the seven study groups.

We identified 638 participants across the entire lifespan (18- to 89-years old) that met the inclusion criteria. One subject was excluded because they were an outlier on the facial ER test (more than 4 standard deviation below the group mean), leaving the total sample to 637. All participants had a normal MMSE score (≥ 25). The participants were divided in seven groups. The characteristics of the study sample are summarized in Table 1. Sex and handedness distributions among the seven study groups were not different (Sex: Fisher’s exact test, *P* = 0.99—handedness: chi-squared test, *P* = 0.72). Moreover, handedness distribution (number of right-handed, ambidextrous and left-handed participants) for our population was similar to what expected in the general population (chi-squared test, *P* = 0.64). The ANOVA analysis showed a significant difference in the ER performance between the seven groups (*F* = 29.795, *P* < 0.001). As data variance was unequal among groups (Levene test, *P* < 0.001), post-hoc analyses were carried out using Games–Howell correction. Post-hoc analysis revealed that the ANOVA effect was driven by reduced performance in participants included in the group 5 (58- to 67-years old), group 6 (68- to 77-years old) and 7 (≥78-years old). Group 2 (28- to 37-years old) was used as control group in the post-hoc analyses as it showed the highest mean score at the test. Bar charts reporting mean, standard deviations and *P*-values for post-hoc comparisons for the seven study groups and scatter plot of facial ER score against age are reported in Figure 1A and B.

### Neural correlates of successful emotion recognition in older subjects

#### Selection of study sample for MRI analysis

Based on behavioural analysis, only participants in the range of age 58- to 67-years old, 68- to 77-years old and ≥78-years old, were included in the analysis to investigate the neural correlates associated with successful ER performance in older subjects. From the 289 participants available for this experiment, 3 were excluded because they did not have all the required MRI sequences (DWI data missing), 2 were excluded because information about qualification level was missing and 1 was excluded because of large ventricles causing registration issues in DWI analysis, leaving the total number of subjects to 283. The characteristics of the MRI study sample are summarized in Table 2.

**Table 2.** Overview of the MRI sample (N = 283) characteristics and outcome measures

Sample characteristics	Mean (±SD) or number (%)	Range
Socio-demographics		
*N*	283	
Age	72.17 (±8.08)	58- to 89-years old
Sex (*N*, % female)	139 (49)	
Educational qualification (*N*, %)		
—No degree	34 (12)	
—GCSE (or equivalent)	26 (9)	
—A-level (or equivalent)	11 (4)	
—NVQ, HND, HNC or other professional qualification	65 (23)	
—CSE or University	147 (52)	
Handedness	80.87 (±47.66)	−100 to 100
ER test		
Total score	98.35 (±14.73)	57 to 119
Reaction time (in ms)	2646.88 (±605.73)	1481.32 to 5327.75
Cognitive and mental health scores		
MMSE score	28.59 (±1.33)	25 to 30
Anxiety score	4.15 (±3.08)	0 to 20
Depression score	2.93 (±2.38)	0 to 12
MRI		
Scanner gradient coil (*N*, before being changed)	51 (18%)	

#### Anatomical scans

**Fig. 2.**
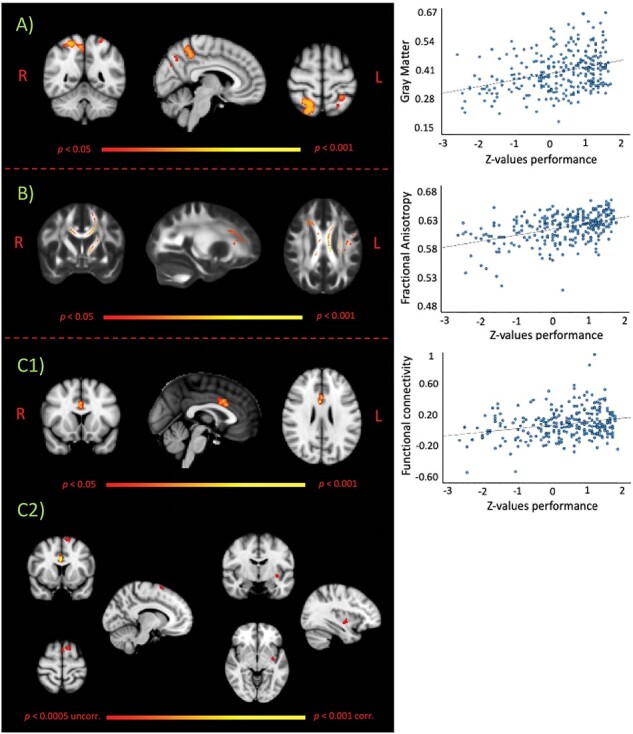
Images depict positive voxel-wise correlations between ER scores and structural and functional MRI images. (A) Voxels showing positive correlation between ER and GM density on VBM analysis were mostly located in the right superior parietal lobule. (B) Voxels showing positive correlation between ER and fractional anisotropy, derived from diffusion-weighted scans, were located in the corpus callosum, extending to left and right anterior thalamic radiation and left inferior fronto-occipital fasciculus. (C1) Positive correlation for seed-based connectivity maps, derived from rs-fMRI images, was observed in the anterior cingulate region. For illustrative purposes, for each of the three correlations reported here, a scatterplot of values extracted from significant regions against ER scores (converted in *Z*-values) is shown. All images displayed here report results with *P* < 0.05, corrected for multiple comparisons. (C2) Using a less stringent threshold (*P* < 0.0005 uncorrected), we also found two clusters in the left superior frontal gyrus and the left insula reflecting positive correlation between ER scores and functional connectivity measures. Red-to-yellow colours define increases in correlation. R, right hemisphere; L, left hemisphere.

After controlling for confounding factors (age, sex, handedness, MMSE, anxiety and depression scores, reaction times at ER test, education level and type of gradient coil), voxel-wise analysis of GM showed that higher ER score was associated with greater GM density. Reporting here and below for each significant difference (i) the cluster size, expressed in number of voxels, (ii) the T-max value for the peak significant area within the reported cluster and (iii) the coordinates of the peak in MNI space, in voxels. Significant voxels were located in the superior parietal lobule bilaterally, mainly on the right side, (1059 voxels, T-max: 4.79, MNI coordinates, *x*–*y*–*z*: 31–34–63) and extending into the precuneus area (Figure 2A). The reported peak of correlation was used to generate a sphere and create the seed for the seed-connectivity analysis described in Section ‘Resting fMRI scans’.

#### DWI scans

After controlling for confounding factors (age, sex, handedness, MMSE, anxiety and depression scores, reaction times at ER test, education level and type of gradient coil), voxel-wise analysis of WM fractional anisotropy (FA) maps showed that higher ER score was associated with greater FA values. Significant voxels were predominantly located in the corpus callosum, extending to left and right anterior thalamic radiation and left inferior fronto-occipital fasciculus (9050 voxels, T-max: 3.96, MNI coordinates, *x*–*y*–*z*: 107–117–108) (Figure 2B).

#### Resting fMRI scans

A total of 20 random subjects across the study groups were identified to create a study-specific training dataset in order to denoise functional images. The optimal threshold for the use of FIX was identified as 10 with a mean (median) true positive rate (TPR) and true negative rate (TNR) of 97.2% (100%) and 85.5% (87%), respectively. This is in line or above the suggested thresholding cut-offs (TPR > 95%—TNR > 70%).

Seed-based connectivity analysis showed that, the seed was correlated with widely spread GM cortical and subcortical regions with very limited or totally absent WM and CSF contamination, demonstrating effective noise removal (Supplementary Figure). Voxel-wise analysis of functional connectivity (FC) maps, controlling for confounding factors (age, sex, handedness, MMSE, anxiety and depression scores, reaction times at ER test, education level and type of gradient coil), showed that higher ER score was associated with greater FC mainly located in the dorsal portion of the anterior cingulate cortex, namely mid-cingulate region (146 voxels, T-max: 4.16, MNI coordinates, *x*–*y*–*z*: 44–68–51) (Figure 2C1). Moreover, using a less stringent thresholding (*P* < 0.0005 uncorrected), we found two clusters showing positive correlation between ER score and FC measures, located in the left superior frontal gyrus and in the left insula (Figure 2C2). Results survived adding GM maps as covariates.

## Discussion

In this study, we have examined ER score variation across the lifespan. Among age-groups showing decline in ER, we have investigated the association between ER performance and MRI-derived measures of brain structure and function, in order to identify those brain regions involved with ER performance maintenance.

### Changes in the ER performance across the lifespan

On a large sample (637 subjects) of cognitively healthy participants (MMSE ≥ 25), covering the adult lifespan (18- to 89-years old), we found an overall age-related reduction in ER performance. Our results are in line with previous meta-analyses and similar studies covering the lifespan (Calder *et al.*, 2003; Sullivan and Ruffman, 2004; Isaacowitz *et al.*, 2007; Ruffman *et al.*, 2008; Malykhin *et al.*, 2023). In particular, we found that ER scores start showing significant decline in the 58- to 67-year-old decade compared to the performance in younger subjects, and they worsen in the last two decades (68- to 77-years old and ≥78-years old). However, our plot (Figure 1B) shows that some subjects across these three age groups can maintain their performance at a similar level of the younger participants. Investigation into the neural characteristics associated with ER scores, described in the following sub-section, may shed light on those brain features and relative functions concurring in maintaining a normal performance despite the effect of age.

### Neural correlates of successful ER in older subjects

Using a multimodal imaging approach, we were able to define structural and functional neural correlates of successful ER in older subjects. Our voxel-wise whole-brain analysis revealed a positive correlation between grey matter measures and ER scores in a cluster of voxels predominantly localized within the right superior parietal lobule, but also including the left superior parietal lobule and extending into the precuneus area. These brain regions are known to be involved in a variety of complex cognitive functions, spanning from attention and visuospatial perception to memory processing and formation (Corbetta *et al.*, 1995; Cavanna and Trimble, 2006). Interestingly, structural, functional and metabolic changes in these regions have been consistently reported in the very early stages of Alzheimer’s disease (Jacobs *et al.*, 2012). We did not find any structural association between ER score and brain regions commonly related to emotional processing (i.e. amygdala) surviving the significance threshold. In support of our finding, a recent study by Malykhin et al., also reported a lack of correlation between amygdala volume and ER performance across the adult lifespan (Malykhin *et al.*, 2023). Our results suggest that in older subjects, ER accuracy is associated with the integrity of brain areas underlying attentional and perceptual processing of the target stimulus, rather than on regions closely related to the affective (limbic) system.

Using a similar statistical approach, but applied to DWI, we found a positive correlation between FA, a marker of WM integrity and ER scores. The association was not only localized in an extended cluster largely placed within the corpus callosum, but also encompassing the left and right anterior thalamic radiation and the left inferior fronto-occipital fasciculus. Interestingly, a previous study found reduced ER performance in patients with callosal agenesis relative to controls, suggesting that reduced callosal integrity may contribute to the development of emotion processing deficits (Bridgman *et al.*, 2014). As the corpus callosum acts as a relay centre, ensuring that different parts of the brain can effectively communicate and send signals to each other, it is plausible to interpret our FA-related findings not so much as impaired ability in recognizing emotions from faces, but rather reflecting reduced connectivity among regions involved in emotional processing.

Seed-based connectivity analysis, using a mask drawn in the right superior parietal lobule (peak of structural correlation analysis), showed that the mid-cingulate (dorsal anterior cingulate) region was positively correlated with ER performance. The mid-cingulate area had been previously shown to be functionally connected with the superior parietal lobule (Hoffstaedter *et al.*, 2014) and, although data in the literature suggest that this brain region is mainly involved in cognitive processes, more recent views have also shown its involvement in emotional functioning (Smith *et al.*, 2019). In particular, evidence suggests that the mid-cingulate area has a central role during emotional conflict resolution (Etkin *et al.*, 2011). As some of the pictures employed in the facial recognition assessment used here, include images that are ambiguous and difficult to interpret, it is possible that participants may need to evaluate the conflicting information during the decision process (Baggio *et al.*, 2012). Two other clusters, reflecting positive correlation between FC measures and ER scores, although at a lesser stringent threshold, also emerged in the left superior frontal gyrus and the left insula. Both these regions have been previously shown in studies investigating emotional functioning processes and associated with affective–perceptual and cognitive–evaluative forms of empathy (Farrow *et al.*, 2001; Uddin *et al.*, 2017).

Taken together our results suggest that normal ER accuracy in older people is associated with preserved GM and WM volumes in cognitive or interconnecting areas, subserving brain regions directly involved in emotional processing.

### Study limitations

Despite the large number of subjects employed in this study to investigate ER variation across the lifespan, and the combined use of structural and functional sequences in identifying neural correlates associated with ER maintenance in older subjects, limitations have to be considered in interpreting our findings. First, we used an ER score based on facial stimuli representing emotions. Although, this is probably the mostly commonly adopted modality to assess this specific ability, there are other ways to present the testing stimuli (i.e. auditory and/or verbal). Future studies should specifically address whether our findings are associated with the specific test we used or can be replicated with other modalities of stimuli presentation. Moreover, the ER assessment employed here was based on static images. This may represent an ecological limitation in the evaluation of the ER ability. Indeed, in everyday life, other people’s emotions are interpreted also considering the environment in which emotions are manifested. Future studies should further investigate into this potential confounder in evaluating ER changes along the ageing process.

### Conclusions

Overall, our behavioural and imaging-based findings offer new insights into the neurobiological mechanisms of successful ER in older people. Moreover, they provide further support to the routine inclusion of ER assessment, along with cognitive evaluation, in older populations. The study of ER ability, combined with imaging techniques, could be crucial for detecting alterations in brain areas associated with a greater risk of developing late-life pathologies or as an early sign of impending neurological disorders. Frontotemporal dementia, Alzheimer’s disease, Parkinson’s and Huntington disease populations have all shown reduced ER performance relative to healthy controls (Gonzalez-Alcaide *et al.*, 2021). Moreover, recent studies have highlighted that even in prodromal forms, such as mild cognitive impairment, ER score was found to be diminished compared to healthy subjects (Park *et al.*, 2017). Decreased ER performance could therefore be an early marker of ‘malfunctional ageing’ process, reflecting the reduced integrity/functionality of the underlying brain areas.

## Supplementary Material

nsad058_Supp

## Data Availability

The data collection and sharing for this project was provided by the Cam-CAN (available at http://www.mrc-cbu.cam.ac.uk/datasets/camcan/). Cam-CAN funding was provided by the UK Biotechnology and Biological Sciences Research Council (grant number BB/H008217/1), together with support from the UK Medical Research Council and University of Cambridge, UK.
