# Supplementary material for: Neural correlates of successful emotion recognition in healthy elderly: a multimodal imaging study
Source: Soc Cogn Affect Neurosci. 2023 Oct 26;18(1):nsad058. doi: 10.1093/scan/nsad058 (PMC10612567; doi:10.1093/scan/nsad058)
Supplement: nsad058_Supp [file nsad058_supp.zip › Supplementary figure.docx]

**Supplementary figure.** Average map of seed-based analysis for the 283 subjects. Overall, the seed (dark blue cluster of voxels) was correlated with widely spread gray matter cortical and subcortical regions (including the amygdala circled in light blue). Voxel-wise analysis of functional connectivity maps showed that higher ER score was associated with greater FC mainly located in the anterior cingulate region (green cluster of voxels, same as Figure 2 C1 in the main text). Red-to-yellow colours define areas of positive correlation with the seed region. All images displayed here report results with *p* < 0.05, corrected for multiple comparisons. R, right hemisphere; L, left hemisphere.
