# Supplementary figures and images for: Neural correlates of successful emotion recognition in healthy elderly: a multimodal imaging study
Source: Soc Cogn Affect Neurosci. 2023 Oct 26;18(1):nsad058. doi: 10.1093/scan/nsad058 (PMC10612567; doi:10.1093/scan/nsad058)

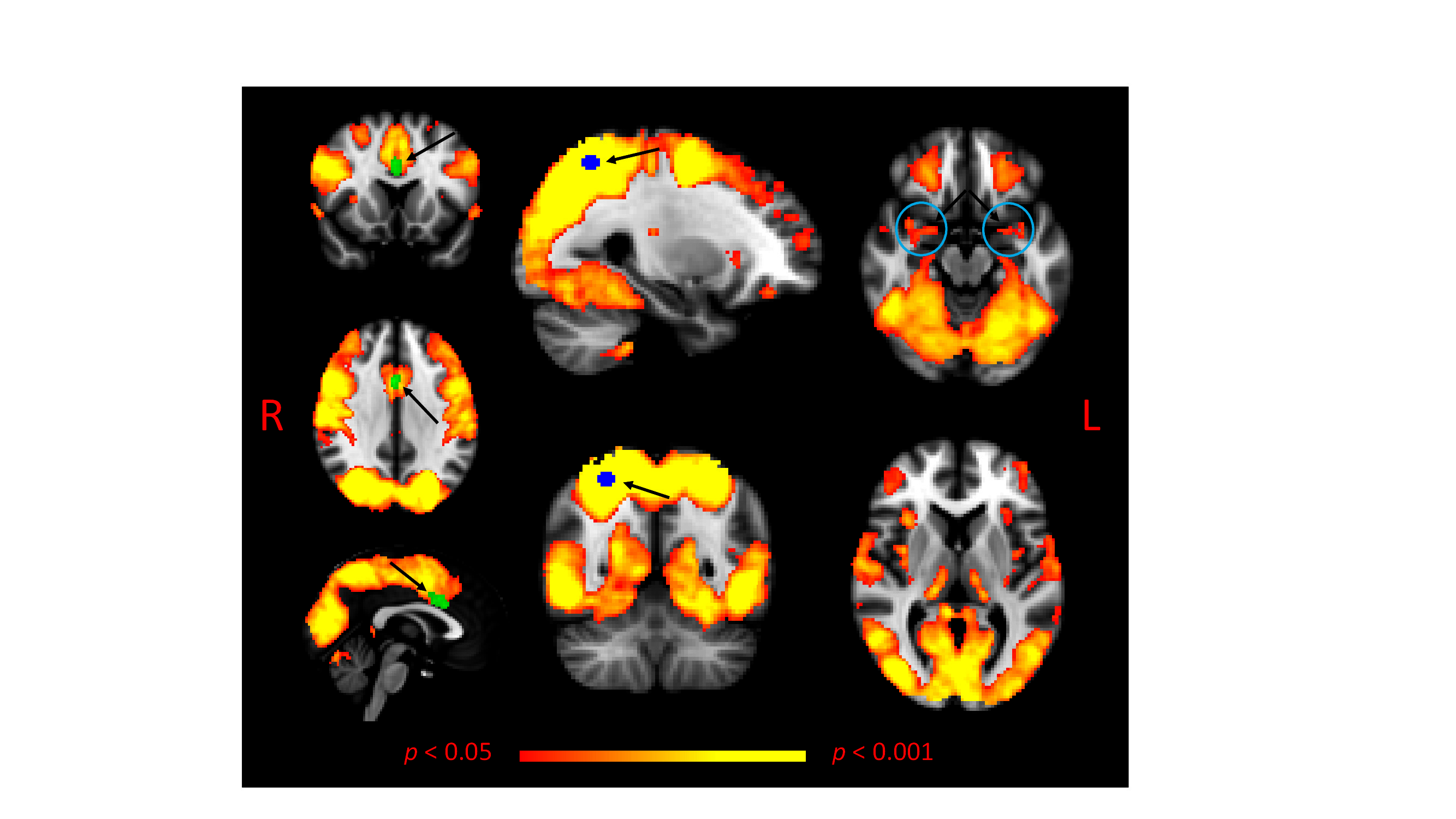

Supplement: nsad058_Supp [file nsad058_supp.zip › scan-23-086-File006.jpeg]
